# Evaluating effects of a new hip-knee-ankle-foot orthosis on gait parameters in healthy individuals

**DOI:** 10.1177/20556683261491668

**Published:** 2026-09-24

**Authors:** Kourosh Barati, Shahrbanoo Bidari, Mohammad Hadadi, Mohammadtaghi Karimi, Mahziyar Darvishi, Amin Komeili

**Affiliations:** 1 Department of Orthotics and Prosthetics, School of Paramedical and Rehabilitation Sciences, 37552 Mashhad University of Medical Sciences, Mashhad, Iran; 2 Orthopedic & Rehabilitation Research Center, Shiraz University of Medical Sciences, Shiraz, Iran; 3 Department of Orthotics and Prosthetics, School of Rehabilitation Sciences, 48435 Shiraz University of Medical Sciences, Shiraz, Iran; 4 Faculty of Kinesiology and Physical Education, 7938 University of Toronto, Toronto, ON, Canada; 5 Department of Biomedical Engineering, 2129 University of Calgary, Calgary, AB, Canada

**Keywords:** Dynamic Balance, Orthotic Devices, Kinematics, Spinal Cord Injuries

## Abstract

**Background:**

Hip-knee-ankle-foot orthoses (HKAFOs) are commonly used to assist gait; however, conventional designs are associated with high energy expenditure and poor mediolateral balance.

**Objective:**

This pilot study examined whether permitting additional motion in selected hip and ankle planes altered joint kinematics and dynamic-balance parameters in healthy participants.

**Study Design:**

A randomized cross-over trial was conducted with 20 healthy participants aged 18-25.

**Methods:**

Gait performance was evaluated under five conditions: conventional HKAFO (locked/unlocked knee), Multiflex HKAFO (locked/unlocked knee), and no orthosis. Kinematic and balance data were collected. Key variables analyzed included center of pressure (CoP) velocity, mediolateral sway, and joint range of motion (ROM).

**Results:**

Compared with the conventional HKAFO configurations, the Multiflex HKAFO permitted greater abduction/adduction and ankle inversion/eversion ROM. With the Multiflex HKAFO and an unlocked knee joint, CoP velocity and mediolateral excursion were not significantly different from walking without an orthosis. However, knee flexion/extension and ankle dorsiflexion/plantarflexion remained restricted relative to walking without an orthosis.

**Conclusion:**

In healthy participants, the Multiflex HKAFO permitted greater motion in selected hip and ankle planes than the conventional design while producing lower center-of-pressure velocity and mediolateral excursion in some configurations.

## Background

Spinal cord injury (SCI) is a significant health concern, with approximately 289,000 individuals affected in North America as of 2012, and an estimated 17,000 new cases annually.^1^ The economic burden per patient is substantial, ranging from $1.5 to $3.0 million for individuals with incomplete paraplegia and complete tetraplegia, respectively.^2^ SCI presents a major challenge to mobility, affecting gait mechanics and overall balance. Individuals with SCI often require assistive devices such as orthoses to compensate for the functional loss, enabling basic ambulation.^3^ Hip-knee-ankle-foot orthoses (HKAFOs) are commonly prescribed for individuals with severe lower limb impairments to provide structural support and facilitate upright walking.^4^ However, conventional HKAFO designs often have several limitations as they can restrict natural joint movement, result in an inefficient gait pattern, and increase the energy cost of walking, ultimately reducing their practicality for daily use.^5^

In particular, the mediolateral instability associated with conventional HKAFOs presents a significant barrier to effective ambulation, requiring users to exert considerable effort to maintain balance.^6,7^ Additionally, traditional designs often incorporate locked joints, restricting knee and ankle range of motion (ROM) and resulting in unnatural gait patterns with limited adaptability to uneven terrain.^8^ As a result, users frequently adopt compensatory strategies, such as applying pressure on the upper limbs or using two crutches to widen the base of support (BoS). However, these adaptations can lead to increased energy expenditure and patient discomfort.^8^

Recent advances in orthotic technology have aimed to overcome these limitations by developing more dynamic designs that closely mimic natural human movement. One such innovation is the introduction of multi-axial joints, designed to replicate the complex motion patterns of the lower limb, particularly at the hip and ankle. These advancements are reported to enable more natural joint movement, enhance balance,^9^ reduce energy expenditure,^10^ and improve user comfort.^11,12^

The present study evaluates the biomechanical effects of a new HKAFO, the Multiflex HKAFO, designed to permit additional motion in selected hip and ankle planes. This novel orthosis features a three-axis mechanism, enabling abduction/adduction, hip internal/external rotation, and ankle inversion/eversion, in addition to flexion/extension at the hip, knee, and ankle joints. Accordingly, the objective of this study was to compare the gait kinematics and dynamic-balance characteristics of the Multiflex and conventional HKAFO designs in healthy participants. Walking without an orthosis was included as a biomechanical reference condition and was not intended to represent a clinical alternative for individuals who require orthotic support.

## Methods

### Participants

The study protocol was approved by the Research Ethics Committee of the Regional University of Medical Science (IR.MUMS.FHMPM.REC.1401.094), and all participants provided written informed consent prior to participation. A total of 48 healthy individuals were initially screened for eligibility with 20 volunteers ultimately meeting the inclusion criteria and enrolling in the study (Table 1). Inclusion criteria included age between 18 and 25 years and a Body Mass Index (BMI) of 18 to 25 kg/m^2^. Participants were excluded if they had any neuromusculoskeletal conditions that could affect normal walking or lower limb function, ensuring that gait assessments were not confounded by underlying health issues.

**Table 1.** Demographic characteristics of the 20 participants (10 male and 10 female).

Variables	Mean	Standard Deviation
Age (Years)	21.7	1.4
Body mass (kg)	67.1	10.3
Height (m)	1.7	0.1
BMI (kg/m^2^)	22.5	2.0

### HKAFO Design Specifications

The study introduced the Multiflex HKAFO, featuring a modular structure, specifically designed to accommodate variations in patient height (Figure 1). The orthosis comprised a pelvic shell, two orthotic hip joint mechanisms, two thigh shells, two single-axis orthotic knee joints, two shin shells, two orthotic ankle joints, and two foot shells. All shells were fabricated from polypropylene thermoplastic material, ensuring durability and lightweight support. Each shell was equipped with two inelastic straps to secure the orthosis firmly to the body. The orthotic hip joint mechanism incorporated a ball-and-socket design, offering three degrees of freedom. The ankle’s dual hinge joints allow motion in the coronal and sagittal planes. The hip and knee joints were connected by a bar constrained to slide along a curved track, which couples transverse plane motion with sagittal plane movements. During hip extension, the bar-and-track mechanism induces an internal rotation, while it generates external rotation during hip flexion. This mechanism facilitates a more natural gait pattern, providing up to 25° of hip internal/external rotation and up to 20° of hip abduction/adduction, depending on the degree of hip flexion/extension (Figure 2). This mechanism enables three-planar motion that closely mimics natural hip joint movements during level walking. Additionally, the transition between standing and walking requires pure hip abduction/adduction, which, according to the mechanism, was initially only achievable in combination with flexion and extension. To overcome this limitation, a hinge joint was integrated into the upper part of the hip mechanism, allowing independent abduction/adduction without affecting flexion/extension, with its movement regulated by a pneumatic cylinder. The conventional HKAFO mirrored the Multiflex design but restricted ankle and hip joint movement in the frontal plane.

**Figure 1.**
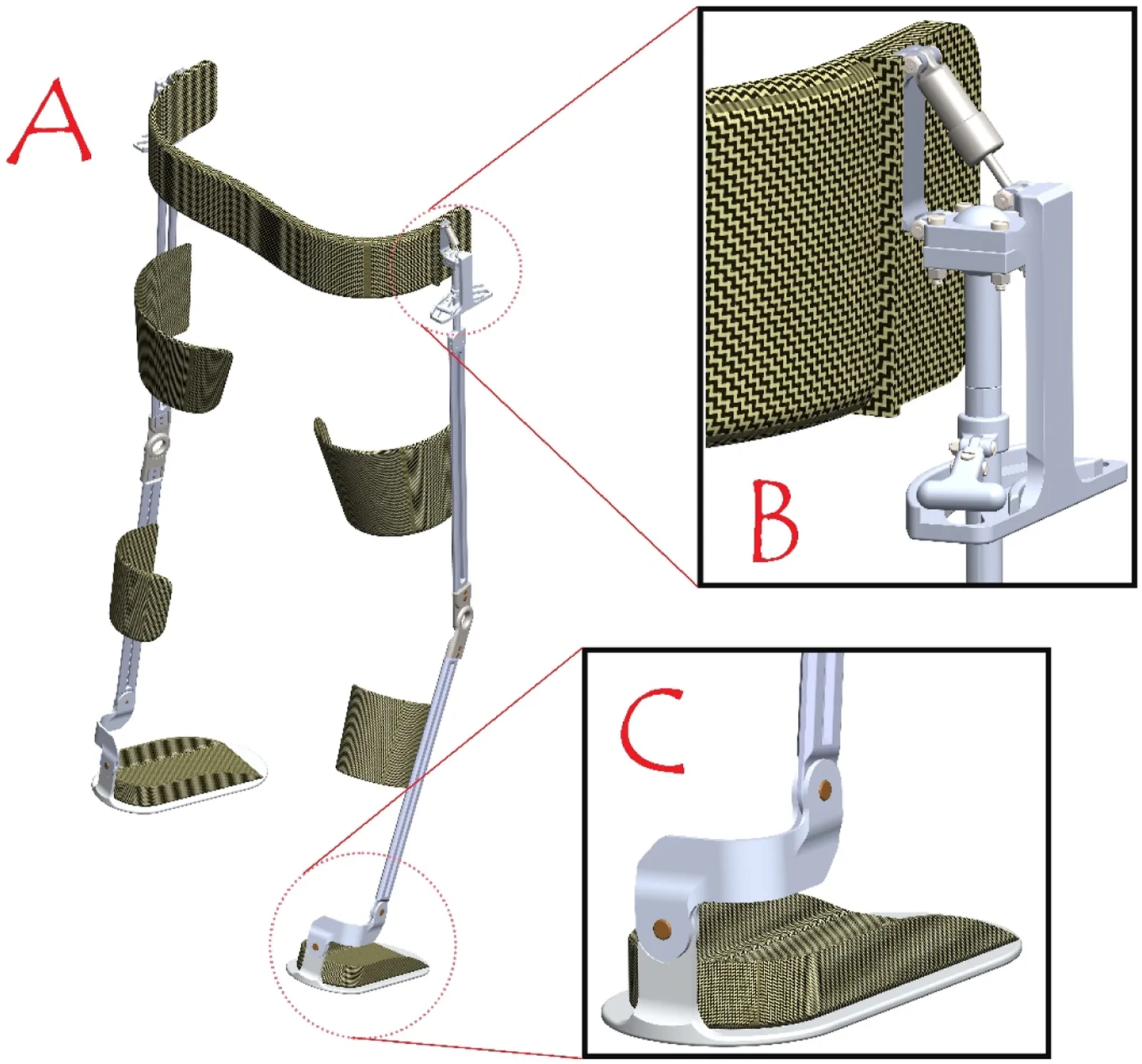
(A) Schematic of the proposed Multiflex Hip-Knee-Ankle-Foot Orthosis (HKAFO) employed in this study. (B) The hip joint mechanism, designed to allow controlled motion in coronal, transverse, and sagittal planes. (C) The ankle joint mechanism, designed to allow free motion in coronal and sagittal planes.

**Figure 2.**
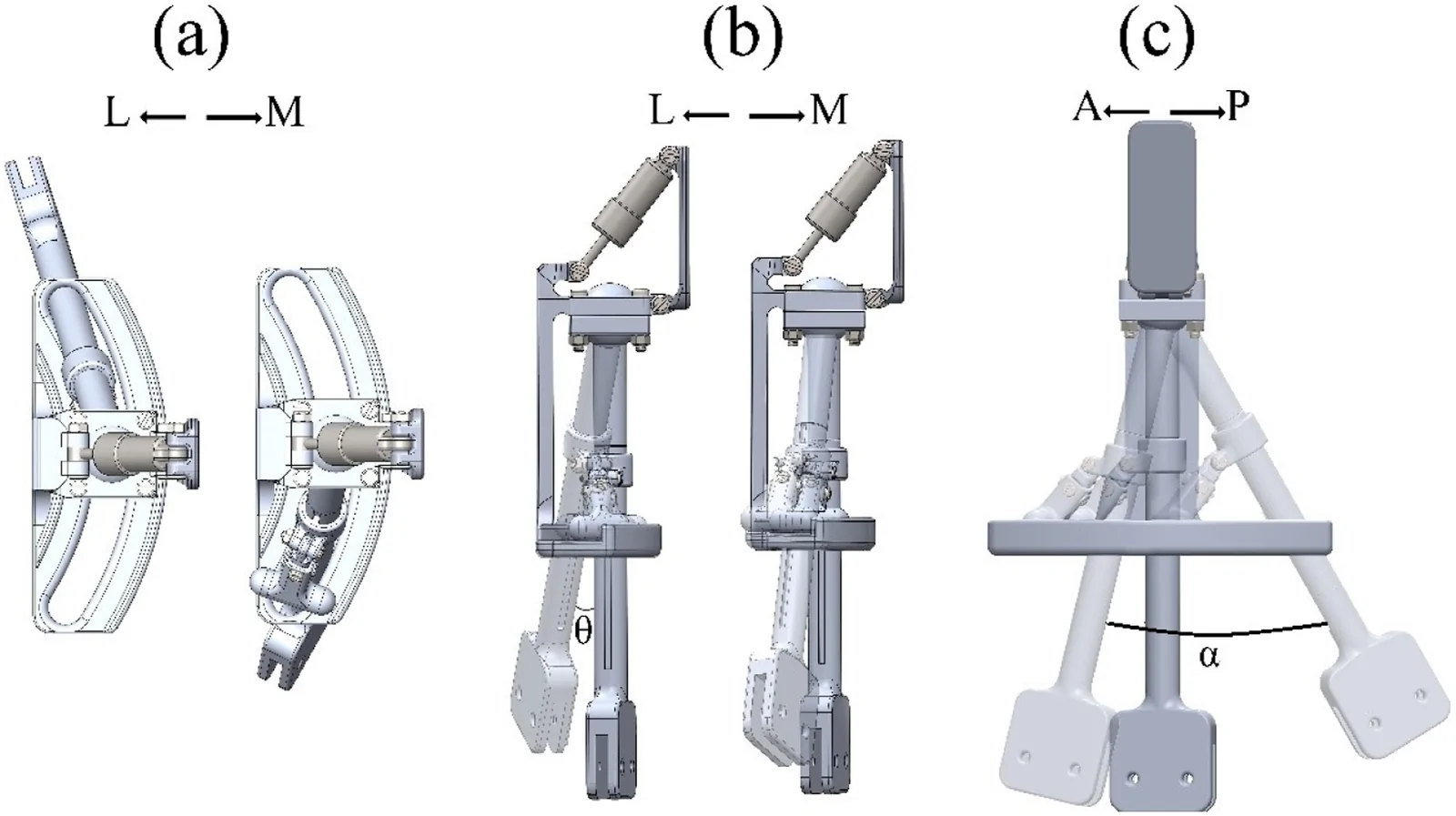
The articulated hip joint mechanism of the orthosis utilized in this study. (A) Range of motion in the transverse plane, illustrating up to 25° of internal/external rotation. During hip extension, the bar-track mechanism induces internal rotation, whereas during hip flexion, it promotes external rotation, mimicking the natural kinematics of human walking. (B) In the neutral frontal plane position, the hip joint maintains a neutral to slightly adducted posture. The track slot enables hip abduction/adduction within θ in 0-20° range, coupled with hip flexion motion. (C) Sagittal plane articulation of the hip joint, demonstrating hip flexion-extension within the range 0 < α < 45° through the bar-track slot mechanism. Additionally, a pneumatic cylinder is incorporated into the orthosis to control pure abduction/adduction movement of the hip joint. L:lateral, M:medial, A:anterior, P:posterior.

### Procedure

This research employed a randomized cross-over design to evaluate the biomechanical effects of the Multiflex HKAFO in healthy participants. Each participant performed walking trials while wearing comfortable walking shoes and was instructed to walk at their self-selected speed along a 12-meter walkway. Gait analysis was conducted under five distinct conditions: 1- conventional HKAFO with a locked knee joint, 2- conventional HKAFO with an unlocked knee joint, 3- Multiflex HKAFO with a locked knee joint, 4- Multiflex HKAFO with an unlocked knee joint, and 5-walking without any orthosis. The order of the five conditions was individually randomized using a blind-draw procedure. Specifically, five numbered balls, each corresponding to one of the experimental conditions, were placed in an opaque bag. Before each condition, the participant selected one ball from the bag with their eyes closed. The selected condition was then performed, and the corresponding ball was removed from the bag before the subsequent draw.

Each condition was repeated until three valid walking trials were obtained. A trial was considered valid when the participant’s entire foot was placed on the force plate during the stance phase. The mean value of the three valid trials was used for subsequent analysis. To minimize learning effects, participants underwent at least 10 minutes of familiarization walking while wearing the corresponding orthotic configuration. Familiarization continued until the participant reported feeling comfortable walking under that condition. Participants then rested for at least 10 minutes and resumed testing only after reporting that they were no longer fatigued. Approximately 20 minutes of rest was considered between the experimental conditions to minimize potential fatigue-related effects. Identical footwear was used across all conditions to eliminate footwear variability. Data acquisition was conducted using a Qualisys motion capture system with six cameras (Qualisys AB, Qualisys Medical, Gothenburg, Sweden), synchronized with a force plate (Kistler, Model 9286B; Winterthur, Switzerland) embedded in the walkway, operating at a sampling rate of 500 Hz (Figure 2). Raw kinematic data were processed using Qualisys Track Manager (QTM) software, which involved marker labeling, gap filling, and filtering of marker trajectories to ensure data integrity. Force-plate signals were retrieved using BioWare software (Kistler 2812A, version 5.3.2.9) and filtered using a third-order Butterworth low-pass filter with a 6-Hz cutoff frequency.

The same anatomical marker locations were used across all experimental conditions. When a component of the orthosis interfered with the placement or movement of a marker, an appropriate opening was created in the corresponding part of the orthosis to maintain the predefined anatomical marker location and minimize interference. Participants did not use walking aids or upper-limb support during any of the walking trials.

### Variables

Dynamic balance parameters included the mediolateral CoP excursion and its velocity. The mediolateral CoP excursion was defined as the maximum displacement of CoP in the mediolateral direction during walking, with larger ranges indicating greater sway. Mediolateral CoP excursion was evaluated during the stance phase. It was calculated as the difference between the maximum medial and maximum lateral CoP positions during stance. CoP velocity was defined as the rate of change in its position, where higher values suggested potentially unstable walking. Kinematic parameters included the ROM of the hip joint in abduction/adduction, flexion/extension, and internal/external rotation; the knee joint in flexion/extension; and the ankle joint in dorsiflexion/plantarflexion and inversion/eversion.

### Data Analysis

OpenSim software (version 4.3)^13^ was employed to simulate movements and predict joint angular positions. A generic full-body musculoskeletal model (Gait2392_Simbody) was implemented, featuring 23 degrees of freedom and 92 muscle-tendon actuators representing 76 muscles in the lower extremities and torso. The model incorporated six degrees of freedom at the pelvis, three degrees of freedom at each hip and ankle, and one at each knee joint. Visual3D software (C-Motion , USA) was used to analyze the motion of foot in three dimensions.

Each participant’s model was scaled to match their anthropometric measurements using skin markers placed over bony landmarks during a static reference trial. Three static poses were analyzed for each participant: one without orthosis, one with the Multiflex HKAFO, and one with the conventional HKAFO. A model was considered “properly scaled” if the Root Mean Square (RMS) error and maximum error of scaled markers were below 2 and 4 (mm), respectively.^13^ In the orthotic conditions, the mass of the HKAFO was incorporated into the model. Inverse kinematics (IK) was then applied to calculate joint angles throughout each walking trial. Because the markers used for inverse kinematics were placed on the participant’s anatomical landmarks rather than on the orthosis, relative movement between the limb and the orthotic device was not independently quantified or explicitly modelled in OpenSim. Accordingly, the kinematic analysis represented the movement of the anatomical segments based on the experimental marker trajectories.

### Statistical analysis

Statistical analyses were performed in SPSS software (version 21; SPSS, Inc., Chicago, IL, USA). The normality of each variable was assessed using the Shapiro-Wilk test, confirming that all variables followed a normal distribution. A repeated measures Analysis of Variance (ANOVA) with one within-subject factor (orthotic conditions) was employed to evaluate each parameter. Upon identifying significant differences between conditions, post hoc analysis was performed using the Bonferroni correction to adjust for multiple comparisons. A p-value of less than 0.05 was considered statistically significant.

## Results

### Hip Flexion/Extension

The mean ROM for hip flexion/extension was approximately 1.8° larger in the Multiflex HKAFO compared to the corresponding condition of conventional HKAFO, though this difference was not statistically significant (p = 0.052). The Multiflex unlocked HKAFO (Condition 4) had a ROM of 35.8°, which was the closest to normal walking without an orthosis (Condition 5), with a ROM of 36.9° (Table 2). Post-hoc analyses did not reveal significant differences among the five conditions.

**Table 2.** Comparison of lower limb joints’ range of motion across five conditions of Multiflex hip-knee-ankle-foot orthosis. Condition 1: conventional HKAFO with a locked knee joint; Condition 2: conventional HKAFO with an unlocked knee joint; Condition 3: Multiflex HKAFO with a locked knee joint; Condition 4: Multiflex HKAFO with an unlocked knee joint; Condition 5: walking without any orthosis. The hip rotation was calculated as the difference between the maximum external and maximum internal rotation angles.

​	Mean (Degree)	Std. Error	95% Confidence Interval		F	P value	Pairwise	Partial Eta Squared	Observed Power
			Lower Bound	Upper Bound					
Hip flexion/extension	​	​	​	​	2.46	0.052	No difference	0.11	0.67
Condition 1	33.5	1.2	30.9	36.1					
Condition 2	34.0	1.4	31.0	37.0					
Condition 3	35.3	1.0	33.1	37.4					
Condition 4	35.8	1.5	32.7	38.9					
Condition 5	36.9	1.8	33.2	40.6					
Hip abduction/adduction									
Condition 1	8.7	0.9	6.7	10.7	15.34	<0.001	1<3,4,5 2<4,5 3<5	0.44	1
Condition 2	9.8	1.2	7.6	12.1					
Condition 3	12.2	0.8	10.4	13.9					
Condition 4	13.9	1.2	11.5	16.4					
Condition 5	15.1	0.9	13.1	17.1					
Hip rotation									
Condition 1	12.2	1.1	9.9	14.4	2.55	<0.05	2<1,3,5	0.11	0.69
Condition 2	9.2	0.7	7.6	10.8					
Condition 3	13.3	1.0	11.2	15.4					
Condition 4	11.8	1.1	8.4	15.2					
Condition 5	11.9	1.2	9.4	14.4					
Knee flexion/extnsion									
Condition 1	8.7	0.7	7.3	10.1	89.07	<0.001	1,3<2,4<5	0.82	1
Condition 2	39.2	2.5	33.9	44.5					
Condition 3	11.2	1.4	8.3	14.1					
Condition 4	38.6	3.3	31.8	45.4					
Condition 5	51.3	3.1	44.8	57.8					
Ankle dorsiflexion/plantarflexion									
Condition 1	19.1	0.8	17.4	20.8	21.14	<0.001	1,2,3<4<5	0.65	1
Condition 2	20.5	0.7	18.8	22.1					
Condition 3	20.1	0.6	18.7	21.5					
Condition 4	22.6	1.0	20.4	24.7					
Condition 5	28.0	1.5	24.8	31.2					
Ankle inversion/eversion									
Condition 1	9.9	0.9	7.9	12.0	44.87	<0.001	1,2<3,4,5	0.70	1
Condition 2	10.2	0.7	8.6	11.9					
Condition 3	17.7	1.0	15.5	19.8					
Condition 4	17.5	1.0	15.3	19.6					
Condition 5	20.1	0.9	18.0	22.1					

### Hip Abduction/Adduction

The mean ROM increased consistently from 8.7° in the conventional HKAFO with a locked knee joint (Condition 1) to 13.9° in the Multiflex HKAFO with an unlocked knee joint (Condition 4). Significant differences in hip abduction/adduction ROM were observed among the conditions (p < 0.001). Post-hoc analysis indicated that the conventional HKAFO with a locked knee joint (Condition 1) exhibited a significantly lower hip Abduction/Adduction ROM than the Multiflex HKAFO with a locked knee joint (Condition 3). Additionally, the Multiflex HKAFO with an unlocked knee joint (Condition 4) demonstrated no significant difference from walking without orthosis (Condition 5) (Table 2).

### Hip Rotation

The total ROM for hip rotation, calculated as the difference between the maximum external and maximum internal rotation angles, ranged from 9.2° in Condition 2 to 13.3° in Condition 3. Statistical analysis revealed a significant difference across the conditions (p < 0.05). A post-hoc comparison indicated that hip rotation in Condition 2 was significantly lower than in Conditions 3 and 5 (Table 2).

### Knee Flexion/Extension

The Multiflex orthosis did not significantly affect knee flexion/extension compared to its corresponding conventional counterpart. However, as expected, the unlocking knee joint mechanism (Conditions 2 and 4) significantly increased ROM compared to the locked knee joint conditions (Conditions 1 and 3). HKAFOs with a locked knee joint exhibited a ROM in the range of 7.0 - 11.2°, which increased significantly to 38.6 - 39.2° when the unlocking mechanism was used. The knee joint ROM in the no-orthosis (Condition 5) was 51.3° which was significantly higher than in all other conditions.

### Ankle Dorsiflexion/Plantarflexion

Significant differences in ankle dorsiflexion/plantarflexion ROM were observed across conditions (p < 0.001). The ROM in Condition 4 (22.6°) was significantly higher than in Conditions 1, 2, and 3, which ranged from 19.1 to 20.1°. The highest ankle dorsiflexion/plantarflexion ROM was observed in Condition 5, with a value of 28.0° (Table 2).

### Ankle Inversion/Eversion

The ROM for ankle inversion/eversion increased significantly in the Multiflex HKAFO(Conditions 3 and 4) compared to conventional HKAFO (Conditions 1 and 2). The ROM progressively increased from 9.9-10.2° in the conventional HKAFO to 17.5-17.7° in the Multiflex HKAFO.

### Dynamic balance

During dynamic balance assessments in walking trials, participants wearing the conventional HKAFO (Conditions 1 and 2) and the Multiflex HKAFO with a locked knee joint (Condition 3) exhibited significantly higher CoP velocity and fluctuation range compared to the no-orthosis condition (Condition 5) (Table 3). In contrast, when wearing the Multiflex HKAFO with an unlocked knee joint (Condition 4), mediolateral CoP excursion and velocity remained comparable to the no-orthosis condition (Condition 5) (Table 3).

**Table 3.** Comparison of the center of pressure parameters (range and velocity) across five walking conditions. Condition 1: conventional HKAFO with a locked knee joint; Condition 2: conventional HKAFO with an unlocked knee joint; Condition 3: Multiflex HKAFO with a locked knee joint; Condition 4: Multiflex HKAFO with an unlocked knee joint; Condition 5: walking without any orthosis. Mediolateral CoP excursion represents the unsigned displacement of CoP in the mediolateral direction. The “<” simbol indicates a significant difference between the compared values.

​	Mean	Std. Error	95% Confidence Interval			F	P value	Comparison	Partial Eta Squared	Observed Power
			Lower Bound	Upper Bound						
mediolateral CoP excursion (mm)										
Condition 1	52.0	3.7	44.0	59.9		24.51	<0.001	3,4,5<14,5<25<3	0.77	1
Condition 2	44.3	3.0	38.0	50.6						
Condition 3	35.4	1.8	31.4	39.3						
Condition 4	30.0	1.9	25.9	34.1						
Condition 5	23.8	1.2	21.1	26.4						
CoP Velocity (mm/s)										
Condition 1	317.2	29.2	255.9	378.5	32.89		<0.001	3,4,5<1,25<3	0.76	1
Condition 2	256.4	22.5	209.2	303.5						
Condition 3	177.1	15.7	144.1	210.0						
Condition 4	140.2	11.4	116.1	164.2						
Condition 5	122.2	7.9	105.7	138.8						

## Discussion

This study aimed to evaluate the effects of a conventional and a Multiflex HKAFO on joint kinematics and dynamic balance during walking in healthy young individuals. Constraint of the hip, knee, or ankle may be necessary to prevent limb collapse during stance, maintain stability, and facilitate forward progression, commonly with an assistive device. The objective of the Multiflex design is therefore not to eliminate joint constraint, but to introduce controlled motion in selected planes without compromising weight-bearing stability. Thus, the Multiflex HKAFO was designed by incorporating abduction/adduction motion into the hip joint and inversion/eversion into the ankle joint. These modifications improved dynamic balance in walking, as indicated by lower mediolateral CoP excursion and velocity values in Conditions 3 and 4 compared to Conditions 1 and 2 (Table 3). These findings indicate differences in dynamic-balance parameters between the Multiflex and conventional HKAFO configurations in healthy participants. These findings have practical implications, particularly given that balance disturbances can significantly increase the energy cost of walking.^14,15^ Because impaired balance control may contribute to the energy demands of walking, the observed improvements provide a rationale for future studies directly measuring metabolic energy expenditure in individuals who use HKAFOs. No conclusions regarding energy expenditure or clinical benefit in individuals with paraplegia can be drawn from the present study.

Allowing hip joint motion, particularly in the frontal plane, is essential for paraplegic individuals to facilitate movement. Moreover, hip flexion is crucial for toe clearance during the swing phase of the gait cycle.^16^ When hip flexion is permitted, stability during the stance phase can be achieved through a backward trunk lean, generating extensor torque at the hip joint, supported by the passive tension in the Y-ligament. This may explain why conventional HKAFOs allow hip flexion/extension. Restricting hip flexion/extension could lead to compensatory lumbar hypermobility, increasing the risk of overuse injuries.^17^ The ROM in hip flexion/extension did not differ significantly across conditions, suggesting that both conventional and Multiflex HKAFOs provide unrestricted hip flexion/extension (Table 2, Figure 3).

**Figure 3.**
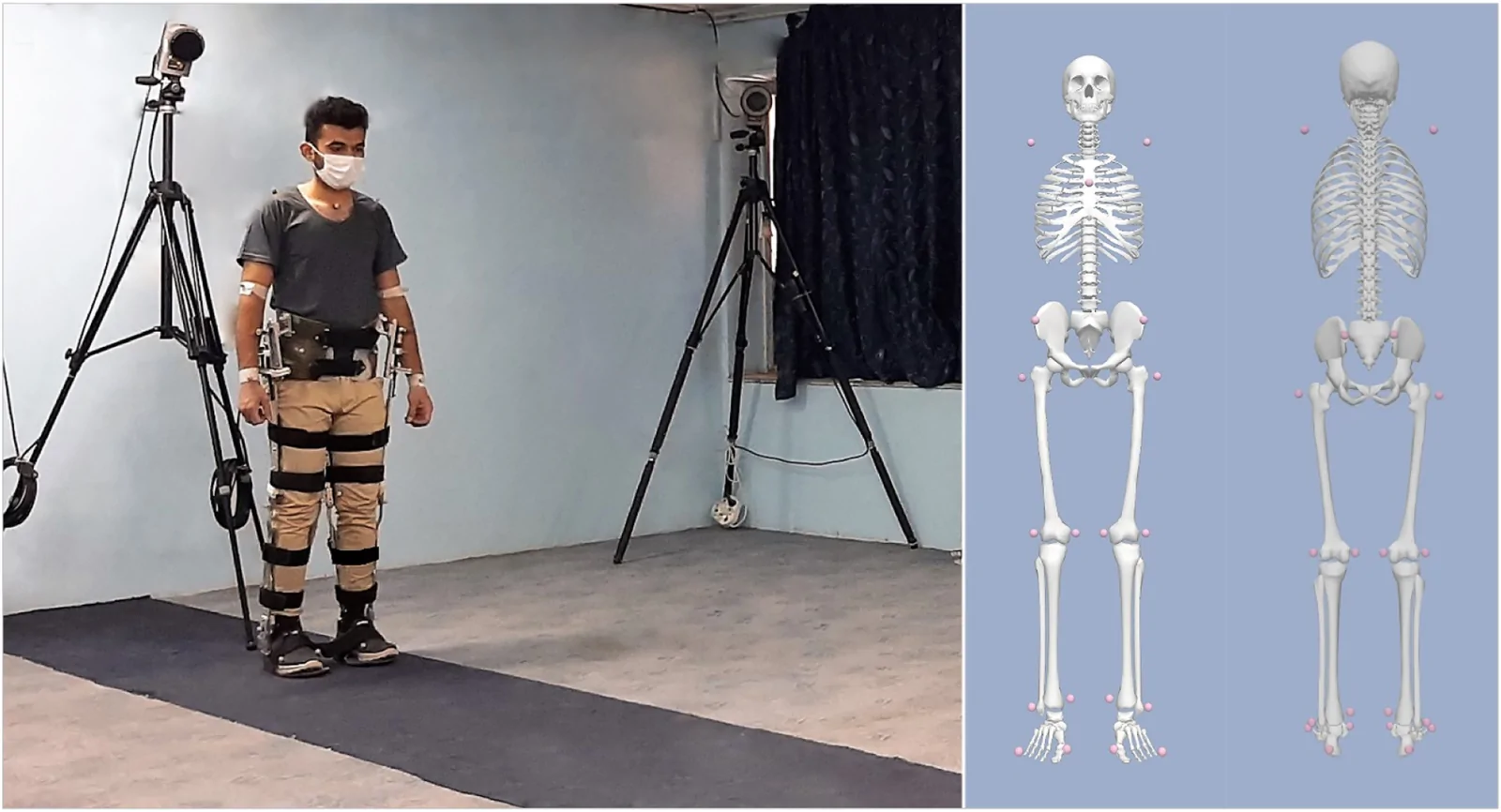
Participants were fitted with 29 reflective markers placed at specific anatomical landmarks, including the sternum, acromioclavicular joints, olecranon tuberosities, wrist styloids, anterior superior iliac spines (ASIS), femoral greater tuberosities, posterior superior iliac spines (PSIS), lateral and medial aspects of the knee joints, medial and lateral malleoli of the ankle joints, calcaneal tuberosities, first metatarsophalangeal joints, and fifth metatarsophalangeal joints.

During walking, the hip joint typically moves within a small arc of 10 to 20 degrees in abduction/adduction,^16^ a movement that is essential for maintaining balance.^18^ When wearing a conventional HKAFO, regardless of whether the knee joint was locked or unlocked, or a Multiflex HKAFO with a locked knee joint, the ROM for hip joint abduction/adduction was significantly reduced compared to walking without an orthosis (Table 2, Figure 3). However, when using the Multiflex HKAFO with an unlocked knee joint (Condition 4, Mean: 13.9°), hip abduction/adduction ROM was not significantly different from walking without an orthosis (Condition 5, Mean: 15.1°). These findings suggest that locking the knee joint in an HKAFO can affect hip abduction/adduction during walking (Figure 3). Therefore, to preserve a normal hip abduction/adduction ROM, allowing knee flexion and extension could be a viable solution, particularly during the swing phase of the gait cycle (Figure 4).

**Figure 4.**
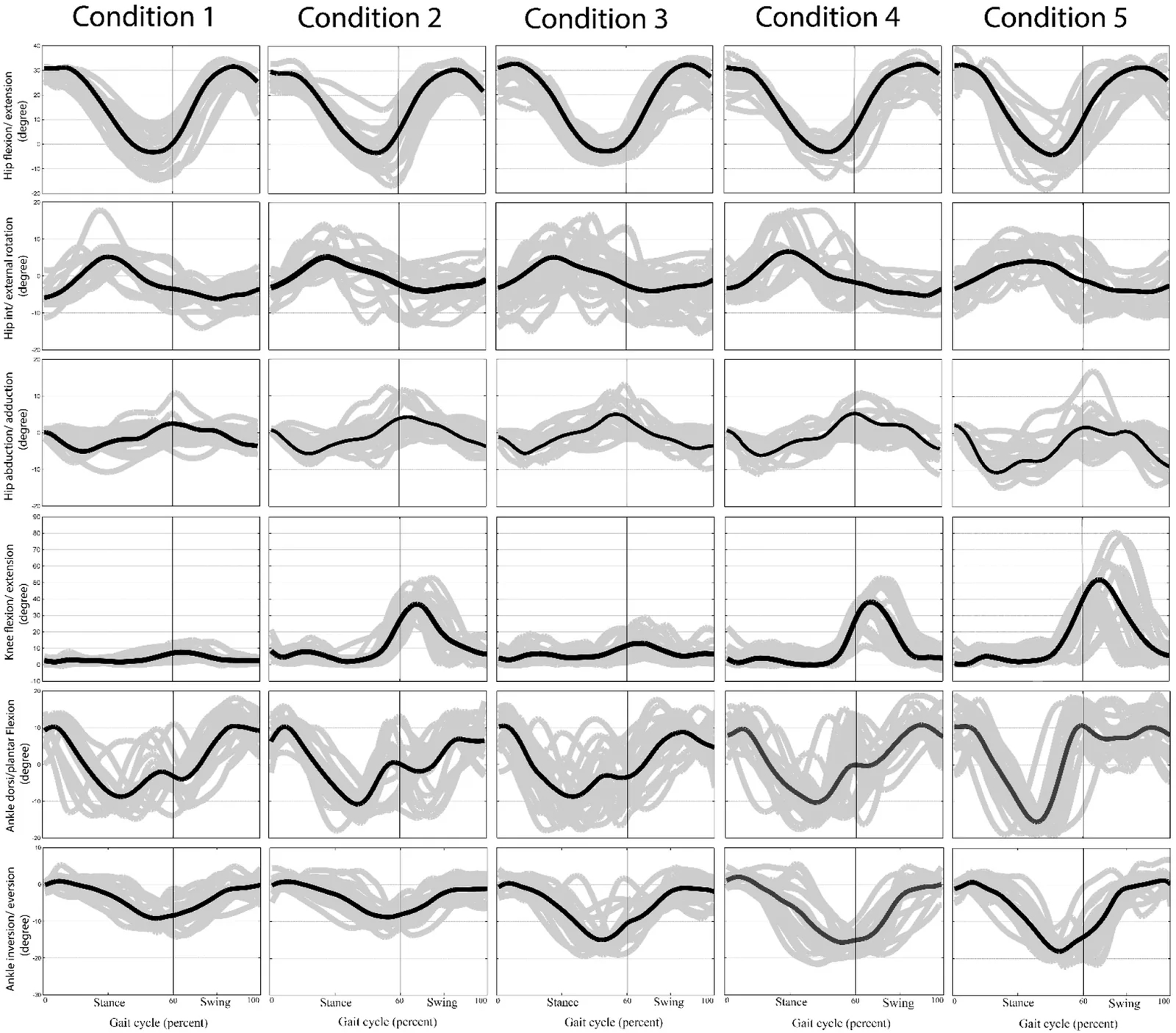
Charts of kinematics of hip, knee, and ankle joints in different conditions. Condition 1: conventional HKAFO with a locked knee joint; Condition 2: conventional HKAFO with an unlocked knee joint; Condition 3: Multiflex HKAFO with a locked knee joint; Condition 4: Multiflex HKAFO with an unlocked knee joint; Condition 5: walking without any orthosis.

The modulation of the center of mass (COM) in the mediolateral direction is significantly influenced by hip abduction/adduction, particularly in challenging balance conditions such as walking and standing on unstable surfaces.^19^ These observations provide a biomechanical rationale for investigating controlled hip abduction/adduction in HKAFO design. However, incorporating this movement into orthotic design presents a movement control challenge, specifically for paraplegic patients. In other words, introducing a ROM in the orthotic joints requires the addition of a motion control mechanism to effectively manage the associated movements. As a result, this increases the complexity, costs, and overall weight of the orthoses. To address this issue, a potential strategy explored in this study involved coupling the abduction/adduction with flexion/extension movements of the hip joint by allowing the hip-knee bar to slide along a track at the hip joint level (Figure 1B). This design provided controlled freedom to the hip joint, which we hypothesized could enhance gait parameters while improving balance control. The success of this design was reflected in greater hip abduction/adduction angles (Table 2) and lower CoP values (Table 3) in Conditions 3 and 4 compared with Conditions 1 and 2.^20–22^

Hip rotation contributes to lower-limb alignment and balance during walking.^23^ Prior research has indicated that a hip rotation ROM of at least 15° is necessary for walking,^16^ making it an important consideration in the design of orthotic hip joints for HKAFO applications. The hip rotation angle in the Multiflex HKAFO was comparable to the no-orthosis condition, ranging from -5° to +6° (Figure 3). However, healthy participants could actively control this additional motion through their intact musculature. Individuals prescribed HKAFOs may have insufficient motor control to regulate transverse-plane hip motion; therefore, unrestricted rotation could compromise lower-limb alignment and stance stability. Clinical application would require an appropriate mechanism to constrain or regulate the magnitude and timing of hip rotation.

Knee flexion plays a crucial role in enabling toe clearance during the swing phase of the gait cycle and facilitating shock absorption during the loading response phase.^16^ The degree of knee flexion is influenced by several factors, including walking velocity and the specific nature of the walking activity, such as level walking, stair descent, or downhill walking. Typically, knee flexion ranges from 35° to 80°.^24^ The ROM for knee flexion/extension was significantly reduced when walking with HKAFOs compared to normal walking, regardless of whether the orthosis was conventional or Multiflex, even when the orthotic knee and ankle joints were unrestricted (Figure 3). Specifically, the ROM for knee flexion/extension while walking with unlocked knee joint HKAFOs (Conditions 2 and 4) was significantly greater than that with locked knee joint HKAFOs (Conditions 1 and 3) (Table 2), but it remained significantly lower than walking without orthosis. The restricted flexion/extension ROM of the participant’s knee joint can be attributed to misalignment between the rotation axis of the orthotic knee joint and that of the participant’s knee. Given concern regarding knee joint stability, unrestricted orthotic knee motion may not be appropriate for individuals with lower-limb weakness or impaired motor control. A suitable control mechanism would therefore be required to permit knee flexion when beneficial while maintaining stability during stance. The present study did not evaluate alternative knee-control strategies, and no conclusions can be drawn regarding the relative suitability of passive, mechanically coupled, or actively controlled mechanisms. It should be noted that because the participants in this study did not have lower-limb weakness or impaired motor control, the present findings cannot determine whether the additional degrees of freedom would provide sufficient stability for individuals who require HKAFOs.

The ROM for ankle joint inversion/eversion was significantly restricted when walking with the conventional HKAFO, regardless of whether the knee joint flexion was locked or unlocked, compared to walking without an orthosis (Figure 3). In contrast, utilization of the Multiflex HKAFO, with either a locked or unlocked knee joint, did not significantly impact ankle inversion/eversion ROM compared to walking without an orthosis (Figure 3, Table 2). Previous research has shown that ankle movement plays a crucial role in fine-tuning COM modulation and facilitating effective foot contact with the supporting surface.^25,26^ Given the lower CoP velocity and mediolateral excursion observed with the Multiflex HKAFO compared with the conventional HKAFO in the present healthy cohort, these findings suggest that permitting controlled ankle inversion/eversion may warrant further investigation as a strategy for supporting mediolateral balance.^16,27^ The addition of degrees of freedom at the hip, knee, or ankle creates control requirements that apply across the Multiflex HKAFO. Although the current prototype uses mechanical coupling and a pneumatic mechanism to regulate selected hip motions, the adequacy of these mechanisms was not evaluated in individuals with impaired lower-limb motor control. Additional motion may require passive stops, locking mechanisms, springs, dampers, mechanically coupled systems, or active control to preserve alignment and prevent collapse during stance. Each approach involves trade-offs among mobility, stability, weight, bulk, cost, power requirements, reliability, maintenance, and ease of donning and doffing. Active or microprocessor-controlled systems may provide adaptable control but could substantially increase device weight, complexity, and cost. Conversely, simpler passive mechanisms may be lighter and less expensive but may provide less adaptability across walking conditions.

This study is subject to several limitations. First, the assessment was conducted on healthy individuals to isolate the biomechanical effects of the Multiflex orthotic joints, independent of actuator performance. This approach aimed to determine whether the redesigned joints, assuming an optimally functioning actuator system, could accurately replicate natural lower limb kinematics. While this methodology allowed for a precise evaluation of joint mechanics, it limits the generalizability of the findings to neurologically impaired populations, such as individuals with paraplegia. Healthy participants possess the muscle strength and motor control required to prevent lower-limb collapse and therefore do not reproduce the stability requirements of individuals prescribed HKAFOs. Future research should focus on integrating actuation systems within the orthotic structure and assessing their impact on gait biomechanics in individuals with motor impairments. Second, this study exclusively involved young participants, a deliberate methodological decision to minimize the potential confounding effects of age-related musculoskeletal degeneration and gait adaptations. While this approach ensured that the observed biomechanical responses were primarily attributable to the orthotic modifications, it limits the generalizability of the findings to older adults or individuals with neuromuscular deficits.

Future research should proceed through sequential engineering and clinical-evaluation stages. First, the mechanisms controlling motion at the hip, knee, and ankle should be refined and evaluated through bench testing to determine their mechanical reliability, range of motion, resistance, and ability to provide adequate stability under weight-bearing conditions. The effects of these mechanisms on device weight, bulk, cost, power requirements, maintenance, and ease of donning and doffing should also be assessed. Subsequent studies should involve individuals who are prescribed HKAFOs, beginning with supervised feasibility and safety testing. These studies should evaluate stance stability, risk of lower-limb collapse and falls, upper-extremity loading, reliance on crutches or walkers, walking speed and endurance, metabolic energy expenditure, user comfort, and perceived exertion. Comparisons should initially be made with participants’ conventional HKAFOs rather than with walking without an orthosis. If safety and feasibility are established, larger and longer-term studies will be required to determine whether the Multiflex design provides meaningful functional or clinical benefits.

## Conclusion

This pilot study provides preliminary evidence regarding the biomechanical feasibility of the Multiflex HKAFO in healthy participants. Compared with the conventional HKAFO, the Multiflex design permitted additional hip abduction/adduction and ankle inversion/eversion ROM and was associated with lower CoP velocity and mediolateral excursion in selected configurations. When the Multiflex HKAFO was used with an unlocked knee joint, CoP parameters were comparable to those observed during walking without an orthosis, although knee flexion/extension and ankle dorsiflexion/plantarflexion remained restricted.

These findings should be interpreted within the context of the study population. Healthy participants possess intact muscle strength and motor control and therefore do not reproduce the stability and mobility challenges experienced by individuals who require HKAFOs. Consequently, the present results demonstrate biomechanical feasibility only and do not establish safety, reduced energy expenditure, clinical effectiveness, or suitability for individuals with paraplegia. Further studies in the intended clinical population are required to evaluate stability, safety, energy expenditure, functional mobility, and user experience before clinical recommendations can be made.

## Data Availability

The data supporting the findings of this study can be obtained from the corresponding author upon making a reasonable request.*
